# Phencyclidine Induced Oculogyric Crisis Responding Well to Conventional Treatment

**DOI:** 10.1155/2015/506301

**Published:** 2015-05-26

**Authors:** Hassan Tahir, Vistasp Daruwalla

**Affiliations:** Department of Internal Medicine, Temple University/Conemaugh Memorial Hospital, 1086 Franklin Street, Johnstown, PA 15905, USA

## Abstract

*Background*. Oculogyric crisis is a form of acute dystonic reaction characterized by involuntary upward deviation of eye ball. Its causes are broad with antipsychotics and antiemetics as the most common causes. *Case Presentation*. A 25-year-old man with the past medical history of marijuana use presented to ED with involuntary upward deviation of eye 1 day after using phencyclidine (PCP) for the first time. He did not have any other symptoms and was hemodynamically stable. All laboratory investigations were normal except urine drug screen which was positive for PCP. Patient was treated with IV diphenhydramine which improved his symptoms. *Conclusion*. Illicit drug abuse is a growing problem in our society with increasingly more patients presenting to ED with its complications. The differential diagnosis of acute dystonic reactions should be extended to include illicit drugs as the potential cause of reversible acute dystonias especially in high risk patients.

## 1. Introduction

Dystonia is a movement disorder characterized by sustained or intermittent muscle contractions causing abnormal, often repetitive, movements, postures, or both. Dystonic movements are typically patterned, twisting, and may be tremulous. Dystonia is often initiated or worsened by voluntary action and associated with overflow muscle activation [1]. Drug induced acute dystonia (DID) is one of the commonest forms of secondary dystonia, along with tardive dystonia. This complication occurs in wide frequency range, depending on the specific drugs prescribed, indications, and studied populations [2]. Clinical presentation commonly includes craniocervical distribution with blepharospasm, buccolingual, mandibular, face and neck dystonia, and oculogyric crisis with contracture of the extraocular muscles leading to conjugate eyes deviation, usually with a predominance of the superior rectus muscle and consequent upward eye deviation [3]. Drug abuse is a rising problem in our society with increasing number of drug abusers being brought to the emergency department because of its complications. Hallucinogens are a class of illicit drugs that cause profound distortion in person's sense of reality. Phencyclidine (PCP), also known as angel dust, is the most dangerous of all hallucinogens due to its effect on behaviour. Unfortunately, there has been a recent increase in the number of emergency visits involving PCP [4]. Rare manifestations and complications of PCP are increasingly seen due to the rising burden of its use [5]. We report a case of 25-year-old man who developed acute oculogyric crisis after using PCP for the first time.

## 2. Case Presentation

A 25-year-old man presented to emergency department with involuntary sustained upward deviation of eyes for one day. According to the patient, he had been using marijuana almost once in a week for the last 5 years but this time he wanted to try a different drug. One day ago, he smoked angel dust with tobacco and also snorted it a little. This was the first time he was using PCP and as per the patient he used very small quantity. After that he felt dizzy and slept whole night. When patient woke up in the morning, he had both of his eyes involuntary deviated in upward direction. His girlfriend immediately brought him to ED for further evaluation. The patient denied any fever, headache, light headedness, slurred speech, weakness, diplopia, and auditory or visual hallucinations. Patient did not have any major medical illness other than marijuana use. He denied any family history of seizures, stroke, or cancer. Patient was not on any medication and also denied any accidental use of antiemetics or antipsychotics recently.

He was hemodynamically stable at the time of his admission. On neurological examination, he was well oriented in time, place, and space. Pupils were equal and reactive to light. Sustained conjugate upward deviation of eyes was noted. The patient was able to bring his eyes back to normal position with forceful effort but eyes used to deviate back to upward position within few seconds. Visual field, visual acuity, and ocular movements testing could not be done due to his upward deviation of eyes. Intraocular pressure was normal and fundoscopy showed normal retina and fundus. Cranial nerve functions were intact, power was 5/5 in all extremities, and there was no sensory loss. The rest of the physical examination was unremarkable. All laboratory data including complete blood count, serum electrolytes, and renal and liver function tests were within normal limits. Urine drug screen was done which came back positive for phencyclidine (PCP). Based on the onset of oculogyric crisis after taking PCP and positive urine drug screen, the diagnosis of PCP induced oculogyric crisis was made. Patient was seen by a neurologist for the evaluation of oculogyric crisis who recommended against any neurological imaging as the case was typical of dystonia and the patient did not have any headache, neck stiffness, seizure, or focal neurological deficits suggestive of intracranial pathology. Decision was made to give IV Benadryl and check response first.

Patient was given 50 mg of diphenhydramine (Benadryl) intravenously once. After 30 minutes, patient's eyes reverted back to normal position. Repeat neurological examination showed equal and reactive pupil. Eye movements were normal in all directions with normal visual acuity and no visual field defect. There was no motor or sensory deficit and his gait was normal. Patient was discharged on 25 mg of Benadryl TID for the next 2 days and also counselled about cessation of illicit drugs. Patient did not have any acute dystonia on follow-up.

## 3. Discussion

Acute dystonia is a movement disorder characterized by intermittent or sustained involuntary muscle contractions involving face, pelvis, trunk, neck, or rarely larynx [6]. Oculogyric crisis is a type of acute dystonia characterized by spasmodic movement of eyeball, usually upward, and each spasm lasts from seconds to hours. Oculogyric crisis is not usually life threatening but it can be very distressing to the patient and family. The causes of acute dystonic reaction are broad with drugs being the most common cause. Rarely, brain stem lesions, encephalitis, and trauma can also cause acute dystonias [7]. Cocaine and ecstasy have also been reported to cause acute dystonic reaction [8]. Although the main mechanism of acute dystonic reactions is still unclear, it is believed that central dopamine blockage with resulting increase in striatal acetylcholine may be the underlying mechanism [9]. Differential diagnosis of oculogyric crisis also includes epilepsy, encephalitis, tetanus, hypocalcemia, brainstem lesions, cystic glioma, and Wilson's disease [10, 11], so it is important to rule out these conditions before making a diagnosis of oculogyric crisis due to drugs. Thorough history, physical examination, and baseline laboratory investigations can help to rule out important differential diagnoses.

Acute dystonic reactions are treated by anticholinergic medications like benztropine, promethazine, or diphenhydramine. Antihistamines like promethazine and diphenhydramine can be successfully used for treating acute dystonias due to their additional anticholinergic effects. Patient is given 2 mg of benztropine or 50 mg of diphenhydramine and watched for improvement of dystonia over the next 15 minutes. This step is both therapeutic and diagnostic. Medication should be preferably given via IV route and in majority of cases, symptoms resolve in 10 mins [12]. In some cases, more than one dosing is necessary for complete resolution of dystonia. In refractory cases or in the presence of contraindications to anticholinergics, diazepam can be used to treat dystonias with variable success [12]. Patient should be followed up for at least 2-3 days, as dystonia caused by long acting drugs may cause relapse of dystonia.

PCP or “angel dust” is a common hallucinogen that is sold illegally in many different forms and is usually smoked with marijuana or tobacco [4]. Depending on route and dose, its effects can last approximately 4–6 hours. PCP, like other hallucinogens, can distort the patient's perception of reality and produces feeling of detachment from environment and self. Delusions, hallucinations, and paranoia mimicking schizophrenia are possible. In extreme cases, seizures and coma can occur [13]. Unlike other hallucinogens, PCP is notorious for causing mood disturbances which can lead to very violent behaviour, thus making it the most dangerous hallucinogen [13]. Long term use can cause dementia, anxiety, and depression. PCP acts on glutamate receptors of brain where it acts as NMDA receptor antagonist and produces its effect. Glutamate receptor has a role in modulating learning, memory, and mood [14].

Our patient came with oculogyric crisis after using PCP for the first time. Interestingly, patient developed acute dystonic reaction after using only small quantities of PCP thus exhibiting idiosyncratic drug response. Patient did not have any hallucinations, delusions, or behaviour problems. CT scan of the head was not done because he did not have any headache, neck stiffness, seizure, or focal neurological deficit. IV Benadryl was given and patient response to it was used as a diagnostic test for acute dystonia. Acute dystonia resolved with Benadryl and patient did not have any symptoms on follow-up next day, after 6 weeks, and then 3 months, so the decision about not doing neurological imaging was considered appropriate. Urine drug screen was positive for PCP but the rest of laboratory investigations were within normal limits. Patient was not on any antipsychotic or antiemetic medication. Patient was given 50 mg of diphenhydramine to which he responded well. The fact that his symptoms were relieved by anticholinergics indicates that pathogenesis of PCP induced oculogyric crisis might be similar to acute dystonias caused by antipsychotics. The exact mechanism is still unclear.

## 4. Conclusion

Acute dystonias including oculogyric crisis have been well known to be caused by antipsychotics and antiemetics. Illicit drugs can very rarely cause similar movement disorders. Due to increasing number of patients with drug abuse being brought to the emergency department, it is imperative to include illicit drugs including PCP in the differential diagnosis of acute dystonias. The management is the same as for dystonias caused by antipsychotics, that is, anticholinergics and reassurance. Our case report highlights the fact that oculogyric crises caused by drugs may be reversible and prognosis may be good.

## Abbreviations

PCP: Phencyclidine
DID: Drug induced acute dystonia
NMDA: N-Methyl-D-aspartate.
